# Arrhythmia mechanism dependent pulmonary vein ablation in paroxysmal atrial fibrillation

**DOI:** 10.3389/fphys.2023.1157338

**Published:** 2023-05-24

**Authors:** Lisa A. Gottlieb, Lukas R. C. Dekker, Ruben Coronel

**Affiliations:** ^1^ Department of Cardiology, Bispebjerg Hospital, University of Copenhagen, Copenhagen, Denmark; ^2^ Electrophysiology and Heart Modeling Institute, University of Bordeaux, Bordeaux, France; ^3^ Amsterdam UMC, location Academic Medical Centre, Department of Experimental Cardiology, University of Amsterdam, Amsterdam, Netherlands; ^4^ Department of Biomedical Engineering, Eindhoven University of Technology, Eindhoven, Netherlands; ^5^ Department of Cardiology, Catharina Hospital, Eindhoven, Netherlands

**Keywords:** ablation, focal arrhythmias, paroxysmal atrial fibrillation, pulmonary vein, pulmonary vein isolation, reentry, trigger

## Abstract

Atrial fibrillation (AF) often requires invasive treatment by ablation to decrease symptom burden. The pulmonary veins (PV) are thought to trigger paroxysms of AF, and ablative PV isolation (PVI) is a cornerstone in AF treatment. However, incomplete PVI, where electrical conduction between the PV and left atrium (LA) is maintained, is curative of AF in a subset of patients. This implies that an antiarrhythmic effect other than electrical isolation between the PV and LA plays a role in AF prevention in these patients. We reason that the PV myocardium constitutes an arrhythmogenic substrate conducive to reentry in the patients with curative incomplete PVI. This PV substrate is amenable to ablation, even when conduction between the LA and PV persists. We propose that PV ablation strategies are differentiated to fit the arrhythmogenic mechanisms in the individual patient. PV substrate modification in patients with PV reentry may constitute a new therapeutic approach that is potentially simpler and more effective, in this subgroup of patients.

## Introduction

In 1998, Haïssaguerre et al. (1998) attributed atrial fibrillation (AF) paroxysms to ectopy from the pulmonary veins (PV). Since, ablative PV isolation (PVI) has been a cornerstone in the invasive antiarrhythmic treatment of patients with paroxysmal AF (episodes of AF shorter than 1 week) in whom pharmacological treatment is ineffective (Calkins et al., 2018). The PVI lesions are created to prevent conduction of ectopic activations from the PV to the left atrium (LA). PV ablation strategies have higher success rate of AF prevention than ablation elsewhere in the atria (Sau et al., 2019). However, the long-term success rate of a single PVI procedure is about 60% (Kis et al., 2017).

Although it has been attempted to completely isolate each PV from the atrium, long-term clinically successful ablation also occurs in paroxysmal AF patients with incomplete isolation or electrical reconnection between the PV and LA (Cappato et al., 2003; Verma et al., 2005; Pratola et al., 2008; Jiang et al., 2014; Kuck et al., 2016). This implies that therapeutic mechanisms other than electrical isolation between the PV and LA play a role in a subset of patients.

In this perspective paper, we elaborate on the idea that the antiarrhythmic effect of incomplete PVI is caused by modification of a local arrhythmogenic PV substrate, rather than sole isolation of ectopic PV foci, at least in some patients. This is in agreement with the current guidelines for AF treatment that point out an improved personalized therapy can be achieved by assessing the pathophysiological processes in the individual patient (Hindricks et al., 2021). We, therefore, do not challenge the therapeutic use of ablative PVI in AF patient but rather argue for a patient-specific mechanism-driven PV ablation therapy. We intend to discuss the basic arrhythmogenic mechanisms likely to occur in the PV (focal and re-entry arrhythmogenesis) and not potential modulating factors, which have been substantially reviewed elsewhere (Nattel, 2017; Linz et al., 2019; Gottlieb et al., 2021b; Gottlieb et al., 2022).

**FIGURE 1:**
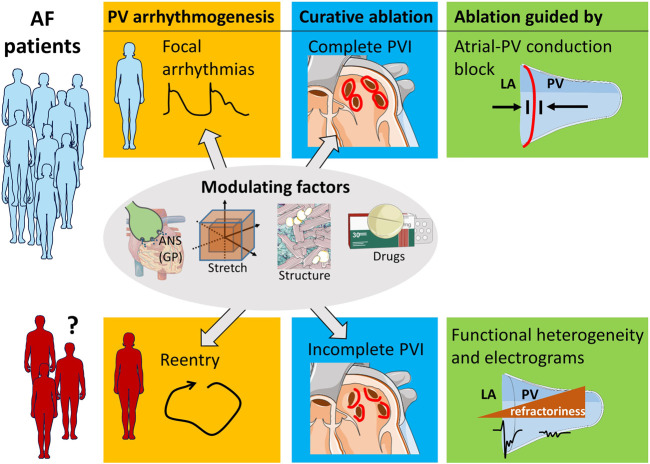
PV ablation strategy dependent on arrhythmia mechanism. Patients with focal arrhythmogenesis in the PV benefit from complete PVI lesions that inhibit atrial-PV conduction. A subset of patients restores sinus rhythm by incomplete PVI. The clinical characteristics of these patients are unknown, and future research can help to identify them. The arrhythmogenic mechanism of their AF likely is reentry involving the PV myocardium. Ablation of this PV substrate can be guided by the morphology of the localized electrograms and by functional properties, such as refractoriness. Modulating factors, such as localized autonomic nervous system (ANS) involving the ganglionated plexuses (GP), atrial stretch, structural remodeling, and pharmacology influence both focal arrhythmogenesis and re-entry.

As of now, we lack the precise knowledge how to select the patients who respond to PVI, to PV ablation, or not at all to AF ablation. Matching the ablation strategy to the paroxysmal AF mechanism in a personalized patient-specific manner may improve patient care (Figure 1).

## PV reconnection and ablation success

Reestablishment of the atrial-PV conduction (reconnection) in one or more PV occurs in the majority of patients after PVI (Jefairi et al., 2019; Daimee et al., 2021). Because recurrence of AF, either early during the 3-month blinding period following the PVI procedure or late (> 3 months), is thought to be caused by re-establishment of electrical atrial-PV reconnection, a new PVI procedure is considered justified in patients with AF recurrence (Haissaguerre et al., 2000; Callans et al., 2004; Gottlieb et al., 2021b). In this case, the reconnected PV are re-isolated. Electrical reconnection also is observed after a second PVI that was performed in reconnected PV following the initial PVI (Cappato et al., 2003). Cappato et al. (2003) even reported that at least 1 PV was reconnected in 3 patients undergoing a fourth PVI ablation procedure. Thus, electrical reconnection after a procedural complete PVI ablation is common.

Several observational studies confirm that stable restoration of sinus rhythm also occurs in patients with electrical atrial-PV reconnection after PVI (Table 1) (Cappato et al., 2003; Ouyang et al., 2005; Verma et al., 2005; Pratola et al., 2008; Jiang et al., 2014; Kuck et al., 2016). Overall, electrical atrial-PV reconnection was present in 110 of 159 patients with an initial, clinically successful ablation (Cappato et al., 2003; Verma et al., 2005; Pratola et al., 2008; Jiang et al., 2014; Kuck et al., 2016). Reconnection had even occurred in all 4 PV in 31% of the patients without AF in one study (Jiang et al., 2014). Moreover, the number of reconnected PV was similar in patients with and without AF recurrence (Pratola et al., 2008; Jiang et al., 2014). Indeed, a meta-analysis including 7 studies conclude that the number of reconnected PV does not correlate with the risk of AF recurrence after PVI (Nery et al., 2016). These observations indicate that the arrhythmogenic mechanisms of AF in a subset of patients are amenable to an ablation strategy that does not prevent focal PV arrhythmias from entering the LA.

**TABLE 1:** Incidence of electrical atrial-PV reconnection after initial PVI and its association with ablation success.

First author (#REF)	PVI strategy	Successful PVI, n	Rhythm monitoring	Re-catheterization (months)	Reconnection, n	Reconnection in 1/2/3/4PV, %
Verma et al. (2005)	Ostial	26	Daily ECG, ECG if symptoms, 48-h Holter ECG (3 + 6 months)	3–6	19% (5/26)	100/0/0/0%
Cappato et al. (2003)	Ostial*	17	24-h Holter ECG (1, 2, + 3 months), ECG if symptoms	5	82% (14/17)	21/79%*
Pratola et al. (2008)	Ostial or WACA	20	24-h Holter ECG (1, 3, 6, 9, 12, 18, + 24 months), weekly ECG: 24–24 months	36	95% (19/20)	5/21/68/5%
Jiang et al. (2014)	WACA	32	24-h Holter ECG (3, 6 + 12 months), ECG if symptoms	12	91% (29/32)	6/31/22/31%
Ouyang et al. (2005)	WACA	7	24-h Holter ECG (3 months), ECG if symptoms, weekly ECG	3–4	0%	NA
Kuck et al. (2016)	WACA	57	Daily ECG, ECG if symptoms	3	75% (43/57)	NA

Re-establishment of the electrical atrial-PV conduction (reconnection) is observed ≥ 3 months after PVI in AF patients with a clinical successful ablation. The reconnection often involves several PV. Asterisk indicates that only the two superior PV were ablated. AF: Atrial fibrillation. ECG: Electrocardiogram. NA: Not applicable. PV: Pulmonary vein. PVI: Pulmonary vein isolation. WACA: wide antrum circumferential ablation.

It can be argued that the clinical outcome of incomplete PVI depends on the arrhythmic activity of the reconnected PV. Thus, reconnection of a PV with lesser arrhythmic activity would play a minor role in AF recurrence. Conversely, development of arrhythmic activity in a reconnected PV could cause ablation failure later on. However, a lower functional arrhythmogenicity in reconnected PV does not seem to be the reason for clinically successful incomplete PVI because randomized trials testing incomplete PVI in all PV have shown similar success rates to complete PVI ablation.

### Randomized trials: incomplete vs. complete PVI

The difference in ablation outcome of complete PVI and intentionally incomplete PVI has been tested in randomized trials (Zhao et al., 2013; Gula et al., 2016; Kuck et al., 2016). Zhao et al. placed 3 radial ablation lines in each PV and observed an increased success rate compared with wide antrum catheter ablation (WACA) PVI (74% vs. 50%, respectively, *p* = 0.025) (Zhao et al., 2013). A success rate of 93% was achieved 6 months after ablation therapy using ablation lines between the PV and the mitral annulus (Kottkamp et al., 2002), whereas similar success rates were achieved between AF patients randomized to either complete WACA or ablation of segments around the PV until bidirectional conduction block (the remaining segments were left non-ablated) (Gula et al., 2016).

Kuck et al. (2016) created incomplete WACA by cessation of radiofrequency energy at the moment of loss of the PV potential at one point in the circular isolation lesion. Clinically successful ablation occurred in both the complete and incomplete PVI groups. Re-catheterization 3 months post-ablation was performed in all patients and revealed that, in the presence of atrial-PV reconnection, the procedural complete PVI was superior in AF prevention compared with procedural incomplete PVI (Kuck et al., 2016). This difference in success rates may also be explained by administration of more ablative energy in the complete than incomplete PVI group (because both groups eventually had PV reconnection) and thereby by extensive modification of the PV.

Thus, complete abolition of atrial-PV conduction is not necessary for restoration of sinus rhythm in all patients. We surmise that this is potentially explained by modification of an arrhythmogenic substrate.

## PV arrhythmogenesis

The arrhythmogenic mechanisms operative in the PV region encompass automaticity, triggered activity, and reentry. The first two mechanisms emanate as “focal” arrhythmias and are sensitive to complete PVI.

### Focal arrhythmogenesis

Solid and direct evidence of automaticity in native PV is scarce but cells staining positive for the ion channel carrying pacemaker current (HCN4) are observed in the PV myocardium of 4 of 5 AF patients in a *post mortem* study and not in 3 patients in sinus rhythm and also not in PV from healthy rats (Yamamoto et al., 2006; Nguyen et al., 2009). The embryonic development of the PV myocardium depends on the transcription factor Pitx2c that suppress automaticity (Rivaud et al., 2021). However, no mutations exist in the PITX2 gene of 96 idiopathic AF patients (Boldt et al., 2010). Instead, a lower level of Pitx2c mRNA in atrial myocytes is associated with recurrence of AF after AF ablation (Reyat et al., 2020). This suggests that automaticity in the PV, if present, may arise from acquired changes in expression profile rather than inborn genetic variations.

Observations of afterdepolarizations and thereby triggered activity in the PV myocardium are limited to animal studies and are scarce in absence of modulating factors, such as autonomic nervous modulation. Chen et al. reported early afterdepolarizations in isolated PV from 7 of 17 dogs, whereas Wang et al. (2003) did not observe early afterdepolarizations in the PV from 50 dogs (Chen et al., 2000).

### Re-entry

For reentrant mechanisms an arrhythmogenic substrate is required. The complex structure in the PV facilitates conduction heterogeneity and together with gradients of refractoriness provide the basis for unidirectional block (Saito et al., 2000; Ho et al., 2001; Hocini et al., 2002; Hamabe et al., 2003; Hassink et al., 2003). Combined, this sets the stage for reentry (Moe et al., 1964; Spach and Dolber, 1986). Although intramural reentry is difficult to document (also experimentally), the PV myocardial sleeve is likely thick enough to sustain an intramural re-entrant circuit (Gottlieb et al., 2021a). Modification of the substrate for reentry does not require complete PVI. A transection of the reentry pathway at any point of the circle suffices (Mines, 1914).

### Arrhythmia mechanism and ablation strategy

The observation that electrical dissociation between the PV and the LA is not necessary for clinically successful AF ablation brings forward the possibility that focal arrhythmias from the PV myocardium are not always responsible for AF arrhythmogenesis and that rapid activations in the PV do not mean that the arrhythmia also is triggered within in the PV. Often, provocative maneuvers, including atrial pacing, have been used to elicit arrhythmias in the PV (Haissaguerre et al., 1998; Haïssaguerre et al., 2000; Haissaguerre et al., 2000). Such premature stimuli can trigger reentrant activations in an arrhythmogenic substrate (Moe et al., 1964). Indeed, premature atrial complexes are common in AF patients and may arise in both the right and left atrium (Schmitt et al., 2002; Cho et al., 2020). Reentry can be the origin of premature beats as well (de Bakker et al., 2002).

Spontaneous focal arrhythmias in the PV likely are the cause of paroxysmal AF in many patients. In these patients, complete long-lasting PVI should be the therapeutic goal. In case of AF recurrence in these patient, re-isolation of reconnected PV should be attempted. On the hand, reentry involving the PV myocardium can also underlie the mechanism of paroxysmal AF in some patients. Patients with PV reentry could be treated by incomplete PVI that modulates the arrhythmogenic substrate. Consequently, a shift in emphasis from isolation of focal PV arrhythmias to ablative modification of an arrhythmogenic PV substrate may be warranted in these selected patients. Tools to single out the AF mechanism in the individual patient need to be developed. Local fractionated potentials recorded from the atrial-PV interface may play a role (Figure 1) (Gottlieb et al., 2021a).

## Hypothesis and perspective

We hypothesize that various arrhythmogenic mechanisms (focal, reentrant) are operative in the PV of patients with paroxysmal AF and ablation strategies directed at the dominant patient specific arrhythmia mechanism can increase ablation success rates.

Identification of the subset of patients with PV arrhythmogenesis by reentry is required (Figure 1). Demographic parameters, such as age and biological sex may play a role in the selection process. Sex-related differences exist in calcium handling, action potential duration, and extracellular matrix structure (Odening et al., 2019). Moreover, the modulating arrhythmogenic factors (body position, obstructive sleep apnea, alcohol dependence, left atrial stretch) can be evaluated before the ablation procedure by history taking, heart rate variability, PV morphology, and mechanics through imaging.

During the ablative procedure, functional properties of the PV myocardium, such as large gradients in refractoriness and conduction heterogeneity may be assessed by an electrophysiological study immediately before delivery of ablative energy.

We showed that the structural composites of the PV myocardium are reflected in the local unipolar electrograms: a large steep deflection and small fractionation corresponded to the larger myocardial mass interspersed with collagen and fat in the proximal PV (Gottlieb et al., 2021a). In addition, arrhythmias inducibility was only possible following pacing in the proximal PV and not in the distal PV, supporting a reentrant mechanism. Hocini and colleagues have similarly shown that electrogram fractionation in the PV myocardium is associated with conduction delay (Hocini et al., 2002). Therefore, ablative modification of the PV substrate prone to reentry guided by the electrogram morphology and repolarization differences may constitute a new therapeutic strategy meriting further research.

Because a critical myocardial mass in combination with the anisotropic structure is needed to accommodate a reentrant activation wave, a critical limit of the extent of ablated PV substrate as antiarrhythmic treatment exists (Garrey, 1914; Allessie et al., 1984). Accordingly, the procedural endpoints of such PV substrate modification by ablation differ from the complete PVI strategy, possibly by including a reduction in electrical activity in the atrial-PV junction and within the PV, instead of conduction block between the LA and PV. Disappearance of local electrogram characteristics may also be a novel end-point. This remains to be tested in AF patients.

Thus, differentiation of the ablation strategy involving the PV based on underlying arrhythmia mechanisms may prove to be beneficial in terms of clinical success rates. Patients with focal PV arrhythmias should have complete PVI without gaps in the encircling lesions, whereas patients with PV reentry may benefit more from ablation targeting reduction of the localized substrate maintaining the reentrant activations than from a conventional PVI.

In conclusion, PV reentry likely underlies arrhythmogenesis in some paroxysmal AF patients, who would benefit more from a targeted PV substrate modification than PVI. A future challenge is to match ablation strategy to arrhythmia mechanism in a personalized patient specific manner, but tools to do this are presently lacking.

## Data Availability

The original contributions presented in the study are included in the article/supplementary material, further inquiries can be directed to the corresponding author.
